# Rift Valley Fever virus M and L genome segment detection: a comparison of field-deployable reverse transcription insulated isothermal PCR (RT-iiPCR) and laboratory-based multiplex reverse transcription real-time PCR

**DOI:** 10.1128/jcm.00430-23

**Published:** 2024-02-02

**Authors:** Jessie D. Trujillo, William C. Wilson, Anthony Craig, Carien Van den Bergh, Thomas Wang, Peter Thompson, Robert Swanepoel, Igor Morozov, Juergen A. Richt

**Affiliations:** 1 Center of Excellence for Emerging and Zoonotic Animal Diseases, Diagnostic Medicine/Pathobiology, Kansas State University, Manhattan, Kansas, USA; 2 Foreign Arthropod-Borne Animal Diseases Research Unit (FABADRU), USDA Agricultural Research Service (ARS), Manhattan, Kansas, USA; 3 Department of Veterinary Tropical Diseases, Faculty of Veterinary Science, University of Pretoria, Vectors and Vector-Borne Diseases Research Programme, Pretoria, South Africa; 4 Research and development, GeneReach USA, Lexington, Massachusetts, USA; 5 Department of Production Animal Studies, Faculty of Veterinary Science, University of Pretoria, Pretoria, South Africa; Mayo Clinic Minnesota, Rochester, Minnesota, USA

**Keywords:** Rift Valley fever phlebovirus, detection, RT-PCR, point-of-need, animals, insulated isothermal RT-PCR, zoonoses

## Abstract

Rift Valley Fever phlebovirus (RVFV) is a mosquito-borne zoonotic pathogen that causes major agricultural and public health problems in Africa and the Arabian Peninsula. It is considered a potential agro-bioterrorism agent for which limited countermeasures are available. To address diagnostic needs, here we describe a rapid and sensitive molecular method immediately employable at sites of suspected outbreaks in animals that commonly precede outbreaks in humans. The strategy involves the concurrent detection of two of the three RVFV genome segments (large and medium) using reverse transcription insulated isothermal PCR (RT-iiPCR) performed on a portable, touch screen nucleic acid analyzer, POCKIT. The analytical sensitivity for both the RT-iiPCR and a laboratory-based L and M multiplex reverse transcription real-time PCR assay was estimated at approximately 0.1–3 copies/reaction using synthetic RNA or viral RNA. The diagnostic sensitivity and specificity of detection of RVFV on the POCKIT, determined using sera from sheep and cattle (*n* = 181) experimentally infected with two strains of RVFV (SA01 and Ken06), were 93.8% and 100% (kappa = 0.93), respectively. Testing of ruminant field sera (*n* = 193) in two locations in Africa demonstrated 100% diagnostic sensitivity and specificity. We conclude that the POCKIT dual-gene RVFV detection strategy can provide reliable, sensitive, and specific point-of-need viral RNA detection. Moreover, the field detection of RVFV in vectors or susceptible animal species can aid in the surveillance and epidemiological studies to better understand and control RVFV outbreaks.

**IMPORTANCE:**

The content of this manuscript is of interest to the diverse readership of the *Journal of Clinical Microbiology*, including research scientists, diagnosticians, healthcare professionals, and policymakers. Rift Valley Fever virus (RVFV) is a zoonotic mosquito-borne pathogen that causes major agricultural and public health problems. Current and most sensitive diagnostic approaches that are molecular-based are performed in highly specialized molecular diagnostic laboratories. To address diagnostic needs, we developed a novel, rapid, and sensitive molecular method using a portable PCR machine, POCKIT, capable of immediate deployment at sites of suspected outbreaks. Here, we demonstrate that field-deployable RVFV detection can provide reliable, sensitive, and specific point-of-need viral RNA detection that could be used for diagnostic investigations and epidemiological studies, and can be performed in the field.

## INTRODUCTION

Rift Valley Fever phlebovirus (RVFV) is a zoonotic mosquito-borne RNA virus belonging to the *Phlebovirus* genus of the *Phenuiviridae* family (1). Originally discovered in the Rift Valley in Kenya in 1930, the occurrence and recurrence of RVFV in many African countries are well documented. In 2000, the first known outbreak of RVFV outside Africa occurred in Saudi Arabia and Yemen, and the virus was likely introduced via imported livestock or mosquitoes following outbreaks in East Africa in 1997–1999. The most recent RVFV outbreaks were reported in 2022 in Burundi and Rwanda; in 2021 in Mauritania, Niger, Kenya, and Madagascar; and in 2020 in Kenya and Mauritania [World Organization of Animal Health (WOAH), event management website: https://wahis.woah.org/#/event-management].

RVFV infections in humans are clinically similar to those caused by two re-emerging high-impact pathogens, Ebola and Zika viruses. Like Ebola, and other emerging/re-emerging hemorrhagic fever viruses, RVFV can cause hepatitis and fatal hemorrhagic fever within days of infection, and it can be transmitted through large droplet aerosols or via accidental puncture through contaminated needles/sharps. Like Zika virus, also a mosquito-transmitted pathogen, RVFV can cause encephalitis and ophthalmitis in humans. RVFV epidemics cause massive fetal death and abortion (abortion storms) in cattle and sheep, where greater than 80% of pregnant animals abort. Fetal deformities are commonly reported (2). Aside from abortion storms, outbreaks of the disease in domestic ruminants are characterized by deaths in young animals, although losses of adults are also reported. Humans most commonly acquire infection from contact with infected livestock tissues, or less frequently the mosquito vector, and RVFV in humans generally manifests as a benign febrile illness. However, illness may become more severe and present as fatal hemorrhagic fever, encephalitis, or ocular involvement (3) (https://www.woah.org/fileadmin/Home/eng/Health_standards/tahm/3.01.18_RVF.pdf).

RVFV was classified by the WHO as one of the top 8 emerging pathogens that “pose the greatest public health risk due to their epidemic potential and/or where there are no or insufficient countermeasures” (https://www.who.int/activities/prioritizing-diseases-for-research-and-development-in-emergency-contexts). This classification for RVFV is due to the severe losses of livestock, increasing fatality rates in humans associated with recent outbreaks, and the lack or limited availability of commercially available vaccines and therapeutics for humans and livestock. Additionally, RVFV is a select agent in the USA, and is ranked as the third most dangerous animal threat by USDA-APHIS after Avian Influenza and Foot and Mouth Disease viruses (4). Intercontinental or global dissemination of RVFV could be perpetuated through animal, human, or vector movement, the intentional release of the pathogen as a bioterrorism agent, or succeptable mosquito vectors which are present on almost all continents (5). Multiagency gap analysis regarding RVFV research and detection identified a profound need for deployable and rapid point-of-need (PON) diagnostic tests to detect and help prevent the global spread of this pathogen as well as aid in the mitigation of potentially devastating public and animal health impacts of it’s spread (6).

The ante-mortem detection of RVFV in clinical samples, fomites and vectors is achieved mainly through the use of molecular assays for one or more segments of the virus genome using reverse transcription real-time PCR (RT-qPCR) in clinical molecular diagnostic laboratories (https://www.woah.org/fileadmin/Home/eng/Health_standards/tahm/3.01.18_RVF.pdf). Virus isolation can also be employed for the detection of RVFV in both ante-mortem and post-mortem samples, albeit with less sensitivity and greater risk for laboratory personnel. Virus detection by RT-qPCR in non-inactivated samples and virus isolation requires expensive BSL3-level biocontainment laboratories with expensive equipment and special technical expertise for the safe handling of high-risk samples. The detection of RVFV antibodies and RVFV RNA in inactivated samples can be performed in lower-level biocontainment laboratories (BSL2 level), but infected animals often succumb to their disease or abort prior to the development of detectable antibodies, rendering serology unsuitable for use early in an outbreak.

To address the need for more rapid and reliable detection of RVFV during outbreaks and to facilitate epidemiological studies in endemic regions, we developed a PON RNA detection method for the RVFV medium (M) and large (L) gene segments using internationally approved RT-qPCR assays as the basis (7, 8). The portable POCKIT Nucleic Acid Analyzer (GeneReach, Lexington, MA, USA) was selected for this work as a field-deployable PCR machine. This device uses reverse transcription insulated isothermal polymerase chain reaction (RT-iiPCR) technology for thermocycling. RT-iiPCR, similar to laboratory-based RT-qPCR, uses two sequence-specific oligonucleotides for complementary DNA (cDNA) synthesis from viral RNA (reverse transcription), followed by cDNA amplification using a DNA-dependent DNA polymerase and specific amplicon detection is performed with a third, sequence-specific, fluorescence tagged oligonucleotide (9, 10). RT-iiPCR mixes are lyophilized, stable at room temperature, and ready to use. Here, we report on the analytical sensitivity and specificity, repeatability, and diagnostic accuracy of PON detection of RVFV RNA using the POCKIT machine and compare it to the reference multiplex (MP) RVFV RT-qPCR assay (11) performed on a laboratory thermocycler using synthetic RNA targets and viral RNA isolated from clinical samples derived from animals in both experimental and natural settings.

## MATERIALS AND METHODS

### Reverse transcription real-time PCR (RT-qPCR)

A multiplex RT-qPCR incorporating World Organization of Animal Health (WOAH) single-plex RVFV M and L segment RNA detection assays (7, 8) with additional S segment primers was validated in 2013. We used this assay with a commercially available one-step RT-qPCR master mix (AgPath, Applied Biosystems) (11, 12) as the reference assay for the present translational study. Additionally, we validated a low-cost 4°C-stable one-step RT-qPCR master mix, qScript XLT One-Step RT-qPCR ToughMix (QuantaBio), designated master mix A, by comparing it with the AgPath OneStep RT-qPCR master mix (Applied Biosystems), designated master mix B (11, 13). Modifications to the assay to accommodate the new master mix include (i) final concentrations of primers and probes in a 20 µL qPCR reaction of 0.4 µM and 0.2 µM, respectively, (ii) the use of 2.5 µL of purified nucleic acid per RT-qPCR reaction, (iii) the inclusion of a 20 min reverse transcription step with 45 cycles for qPCR offering improved assay sensitivity, and (iv) a positive Ct cut-off of 38 for all gene segments. Real-time thermocyclers used varied during this project due to the completion of the work in multiple locations and laboratories and included the CFX 96 (Bio-Rad), the MX (Stratagene), and the Step-One Plus or the 7500 (Applied Biosystems). Equipment equivalency was determined using standard curve analysis and validation of positive controls before testing samples at collaborative research sites. Similar sensitivity and reproducibility were seen with each thermocycler, and any intra-assay and instrument variation was corrected by using standardized quantitative *in vitro* transcribed (IVT) RNA controls for the RVFV M and L segments during each RNA extraction and RT-qPCR run. RT-qPCR on clinical samples was performed using triplicate qPCR wells and qPCR master mix A (Quanta master mix). Samples were marked as positive for a specific gene when 2/3 RT-qPCR replicates reported a Ct ≤ 38. A Ct cut-off of 38 was the determined analytical sensitivity and clinical sensitivity of the RT-qPCR assays using known positive clinical samples. For diagnostic sensitivity analysis, both RVFV M and L assays needed to be positive for a sample to be classified as positive. All RT-qPCR assays were performed using appropriate quantitative RT-qPCR positive controls (IVT L/M RNA) and no template controls (NTCs).

### Reverse transcription insulated isothermal PCR (RT-iiPCR)

Validated and currently employed WOAH-approved single-plex RT-qPCR assays for the detection of the RVFV L gene segment (7) and the RVFV M gene segment detection (8) were selected for translation onto the PON platform (Table 1) to provide a high-confidence, dual-gene target detection strategy for RVFV. The modifications to the RT-iiPCR platform included a reduction of the size of the PCR amplicon generated and the use of a minor-groove binder quencher on the probe to increase the melting point differential of the primers and probes. Before testing field samples, performance characteristics of the modified oligonucleotide sets as single-plex assays (one gene detection per reaction) were tested on the PON device using RVFV squence specific DNA, *in vitro* transcribed RVFV RNA and purified RVFV viral RNA, and, finally, RVFV RNA purified from clinical samples (serum) collected from experimentally infected animals. The RT-iiPCR reagents provided by the manufacturer (GeneReach) were quality-controlled, and the initial performance was assessed by the manufacturer. Prepackaged lyophilized master mix in 0.5 mL tubes containing all necessary PCR reagents and the 2× high salt buffer were provided for rehydration of the RT-iiPCR reagent pellet. Five microliters of purified nucleic acid was added, and the entire volume (50 µL) was transferred to the RT-iiPCR capillary tube (R-Tube). R-Tubes were sealed with an insulating rubber cap, briefly centrifuged or flicked to remove air bubbles, and then placed into the deployable PCR device (POCKIT). FAM was detected at a wavelength of 520 nm and the RT-iiPCR run was initiated via the instrument touch screen. The POCKIT device is supplied with a Cubee mini-centrifuge and two pipettes in a portable hard-sided suitcase as the POCKIT Express Kit. In the POCKIT, the RT-iiPCR reaction mix in the R-tube is heated at the bottom, and natural Bénard convection occurs driving fluid cycling through temperature gradients in the column (R-tube), allowing for reverse transcription and PCR steps (denaturation, annealing, extension, and probe hydrolysis) to occur at different zones within the R-tube. Amplicon detection is via the binding and hydrolysis of a target sequence-specific fluorescent-labeled oligonucleotide probe (9). Upon completion of the run, the equipment software analyzes fluorescence before and after the run and provides a non-quantitative interpretative result to the end user, in the form of plus or minus symbols on the device screen. Raw data are collected onto a data storage card that can be viewed and archived in Microsoft Excel. A fluorescent ratio of greater than 1.5 is reported as positive by the device’s internal computational algorithm that is applied to raw fluorescence data from photographs of the capillary tube taken before and after the run. Positive control DNA plasmid was provided by the manufacturer and is comprised of a 736 base pair (bp) fragment of the MP12 RVFV vaccine strain containing sequences for both assays cloned into pGH plasmid (GeneReach, USA). This positive control was used as a field-stable DNA control and was run alternately with a NTC with each alternating POCKIT run during the testing of field samples. Purified RVFV MP12 RNA or quantitative IVT RNA served as a reverse transcription reagent control and was run daily as a reagent test control. Clinical samples were tested on POCKIT with single replicates for the detection of RVFV M and L genes and are performed independently as single-plex reactions. Moreover, there is a variable concentration of RVFV L and M gene present in experimental and field samples. Therefore, the analytical and clinical sensitivity and specificity for the detection of RVFV M and L gene segments on the POCKIT for experimental samples were determined and reported separately for the L and M genes and for dual-gene detection (Table 5),

**TABLE 1:** DNA oligonucleotide primers and fluorescent hydrolysis probes used in the reference multiplex RT-qPCR assays for the detection of RVFV L and M gene segments and in RT-iiPCR assays on the portable device, POCKIT

RT-PCR assay	Name	Sequence (5′ → 3′)	bp^*a*^ start	bp^*a*^ stop
RVFV-M RT-qPCR	RVFV-MF	AAAGGAACAATGGACTCTGGTCA	1,164	1,186
(8)	RVFV-MR	CACTTCTTACTACCATGTCCTCCAAT	1,258	1,233
	RVFV-MPROBE	FAM-AAAGCTTTGATATCTCTCAGTGCCCCAA-BHQ3	1,204	1,231
	Amplicon size	94 bp		
RVFV-L RT-q PCR	RVFV-LF	TGAAAATTCCTGAGACAGATGG	2,908	2,929
(7)	RVFV-LR	ACTTCCTTGCATCATCTGATG	2,997	2,977
	RVFV-LPROBE	HEX-CAATGTAAGGGGCCTGTGTGGACTTGTG-BHQ3	2,946	2,973
	Amplicon size	89 bp		
RVFV-M RT-iiPCR	RVFV-M-F	AAAGGAACAATGGACTCTGGTCA	1,164	1,186
	RVFV-M-R	TTGGGGCACTGAGAGATATCAA	1,231	1,210
	RVFV-M-PROBE	FAM-CTTTTGAGCTCCCTCTT-MGB	1,207	1,191
	Amplicon size	68 bp		
RVFV-L RT-iiPCR	RVFV-L-F	AGGGCTCGGAAGCAATGTAA	2,939	2,953
	RVFV-L-R	GCCTTGGTTCCACTTCCTTG	3,008	2,989
	RVFV-L-PROBE	FAM-CACAAGTCCACACAGGC- MGB	2,973	2,957
	Amplicon size	75 bp		

^*a*^ Sudan 28-2010. GenBank accession#JQ820486.1 (RVFV L gene segment) #JQ820491.1 (RVFV M gene segment).

However, since both experimental and field samples with low viral copy numbers can yield variable RT-qPCR results for each gene target and given that the objective of this testing and assay translation to a portable PCR device was to have high confidence in testing results we applied dual-gene detection, to determine of clinical diagnostic sensitivity and specificity for field samples and included only those samples that were RT-qPCR-positive using the reference laboratory thermocycler for both the RVFV M and L segments (Table 6). Sensitivity and specificity, 95% confidence intervals and predictive values, and Cohen’s kappa were determined using an online calculator (http://vassarstats.net/).

### Quantitative synthetic RNA controls

**Fig 1:**
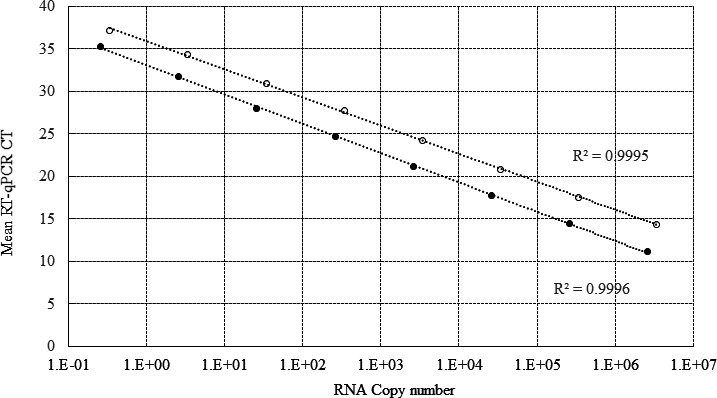
RVFV quantitative RT-qPCR standard curves generated using log_10_ dilutions of quantified *in vitro* transcribed RNA and master mix A. Data represent the mean of nine replicate PCR wells using the RVFV multiplex RT-qPCR method for L and M segment detection. Open circles, RVFV L IVT RNA; closed circles, RVFV M IVT RNA.

*In vitro* transcribed RNA was generated using the T7 transcription kit (MEGAscript, ThermoFisher) from a PCR-generated amplicon cloned in a plasmid (described below). We used an oligo-free RT-iiPCR mix (GeneReach, USA) as well as T7 promoter and terminator primers (Integrated Technologies) in a 9-hour reaction at 37°C in the Bio-Rad CFX 96 thermocycler. The RVFV L plasmid (provided by Hana Weingartl) contains 3,482 bp of the L segment of RVFV (nucleotides 1–3,482 of the ZH-501 RVFV strain), cloned into the pGEM-T plasmid. The RVFV M plasmid (provided by Connie Schmaljohn from USAMRIID) contains 1,451 bp of the M segment of RVFV (480–1,931 of the ZH-501 strain), cloned into the pET-30 Ek/lic plasmid. IVT RNA was DNAse (ThermoFisher Scientific)-treated 3×, column-purified (MEGAclear, ThermoFisher), and confirmed to be DNA-free with qPCR using FastMix II PCR master mix (QuantaBiosciences), and the respective RVFV M and L primer and probe sets at the working dilution. DNA-free IVT RNA was quantitated with spectrophotometry (Cubit, Qiagen), and the copy number was calculated using an online calculator (http://scienceprimer.com/copy-number-calculator-for-realtime-pcr). Tenfold serial dilutions of IVT RNA (5 × 10^6^–5 × 10^−1^ copies) were utilized to generate an eight-point standard curve using nine PCR well replicates per dilution in multiplex RVFV RT-qPCR, performed as three scientific replicates on the CFX laboratory thermocycler (Bio-Rad) (Fig. 1). The copy number (*x*) was calculated using the PCR-determined mean Ct (*y*) for the M and L segments and the slope (*M*) and intercept (*B*) of the RVFV L and M segment IVT RNA standard curve using the formula *y* = *Mx* + *B*. Data are reported as the RT-qPCR-determined copy number (CN) per reaction.

### Virus stocks

The wild-type RVFV strains SA01-1322-2001 (SA01) and 128B-15 Kenya-2006 (Ken06) isolated in Saudi Arabia in 2000–2001 (SA01) and Kenya in 2006–2007 (Ken06-128) (14), respectively, were provided by B. Miller (Centers for Disease Control, Fort Collins, CO). MP12, a modified live vaccine strain, was provided by Dr. William Wilson, USDA. Wild-type viral stocks were propagated in the C6/36 *Aedes albopictus* cell line as previously reported (14) under BSL3 conditions (11). RVFV MP12 was provided by USAMRIID and was propagated once in normal fetal lung fibroblast (MRC-5) cell cultures with Dulbecco's modified Eagle medium (DMEM) and 10% fetal bovine serum (Atlanta Biologicals) under BSL2 conditions. Cells were infected using a multiplicity of infection of 0.01, and RNA extractions were performed when approximately 80% of cells showed cytopathic effect. Total RNA was prepared using TRIzol phase separation followed by ethanol precipitation (11). The RVFV Smithburn vaccine strain (provided by Leonard Ateya at Kenya Agricultural and Livestock Organization, Nairobi, Kenya) was inactivated in ATL buffer (Qiagen, Germany) on-site with heat (80°C for 5 min) and processed using a magnetic bead processor as described below under BSL2 conditions.

### Clinical samples

#### Experimental infections

Archived sera collected from sheep (*n* = 13) and cattle (*n* = 15) experimentally infected with wild-type RVFV SA01 or Ken06 isolates and mock-infected control animals (n= 4) were used (cohort 1) (13–15). Daily sera collected from D0–D6 post-infection (dpi) were TRIzol-LS-treated (Life Technologies) and processed in a standard phase separation protocol using 1-bromo-3-chloropropane and a modified magnetic bead capture protocol (MagMAX-96 Total RNA Isolation Kit, Life Technologies). Briefly, 100 µL of the aqueous phase was added to 90 µL of isopropanol and 10 µL RNA binding bead mix (Beckman) followed by two washes with buffer A and two washes with buffer B followed by the elution of RNA into 50 µL of elution buffer. Extractions were performed using the automated magnetic bead processor (KingFisher 24, Applied Biosystems).

#### Field samples

Archived goat sera (*n* = 50) originating from Washington State, USA, were utilized to determine the specificity for RVFV M and L segment detection (cohort 2). Sheep, cattle, and goat serum samples (fresh and archived) collected in Kenya (cohort 3) and South Africa (cohort 4) were processed as fresh or frozen samples. In the absence of overt RVF disease, attempts to obtain virus-positive samples consisted of testing batches of livestock sera that included specimens found positive during surveillance for the occurrence of RVFV IgM antibodies or seroconversion in endemic areas. An exception was the testing of antelope samples in South Africa where the seroprevalence of RVFV antibody was unknown at the time of testing. Magnetic bead extraction of total RNA was performed using the GeneReach total NA extraction reagents (with modification) on several different automated bead processors: (i) the TacoMini, manufactured by GeneReach, USA, which is a portable magnetic bead processor that can process eight samples per run in a mobile laboratory or in the field, (ii) the Life Technologies, KingFisher 24 and (iii) the Qiagen, Biosprint 96 which are larger-scale magnetic bead processors for use in a laboratory setting. Instruments used for processing clinical samples were dependent on the testing location. Equivalency for various extraction equipment was determined using the performance analysis of validated extraction controls prior to and during the testing of samples. For sample processing, 75–100 µL of serum (heat-inactivated) was added to 350 µL of lysis buffer and 25–50 μL of magnetic beads, followed by the addition of 100–150 μL of molecular-grade isopropanol. Beads were then washed 2× with wash buffer A followed by washes with wash buffer B and molecular-grade ethanol as the final wash. Wash volumes ranged from 200 to 450 μL, depending on the volume of beads used in the extraction. RNA was eluted into 50 µL of elution buffer. This IBC protocol was approved at KSU (#1099).

## RESULTS

### Analytical sensitivity of the RVFV multiplex RT-qPCR assay

The RVFV reference RT-qPCR assay was evaluated using quantified IVT RNA of the RVFV M and L RNA segments to establish the dynamic range and analytical sensitivity of the assay and the performace valitated using quantitative positive RNA controls for the testing of clinical samples. The alternate low-cost, single-tube, refrigeration-stable, one-step RT-qPCR master mix was also evaluated (master mix A, Quanta BioSciences) for translational work in Africa (12). The original RT-qPCR master mix requires storage of the reverse transcription enzyme at −20°C as a separate addition at the RT-qPCR setup (master mix B, Applied Biosystems) (11). RT-qPCR master mix A was tested side-by-side and contains a reverse transcription enzyme incorporated into the master mix that is stable at 4°C. Primers (forward sense and reverse antisense) and detection probe sequences (sense) for the RT-qPCR assay and the specific detection of the RVFV M and L RNA segments in the RT-iiPCR are provided in Table 1. RVFV M RNA detection utilizes a FAM-based fluorescent signal, and the L gene detection uses a HEX fluorophore for gene detection. Results of testing 10× serial dilutions of IVT RNA using both master mixes are summarized in Table 2 and demonstrate equivalency of detection of both the RVFV M and L RNA segments using either RT-qPCR master mix. Single-plex data are presented, and similar results were observed using multiplex RT-qPCR (data not shown). The limits of detection in 100% of RT-qPCR replicates (the LOD_100_) for RVFV M RNA using master mix A or B lies between 35.6 and 37 mean Ct (0.2 RNA copies per reaction). For RVFV L gene detection, both master mixes have an LOD_100_ at approximately 36–37 Ct (0.34 RNA copies per reaction). Notably, both detection assays, when performed in the single-plex or multiplex mode, using either master mix, have an LOD_50_ (50% positive replicates), with a mean Ct of 35 but can detect very low-level RNA at 38 Ct. Master mix A was selected as the primary master mix due to its performance, ease of use at international sites, and reduced cost that will aid in better translation of this testing to low-resource countries. Fig. 1 presents reference standard curves, which plot log_10_ serial dilutions of IVT RNA, which allows the determination of RNA CNs. Utilizing this method for RNA copy number quantification, RVFV L and M RNA CNs were determined in virus stocks and all positive samples. The standard curve regression line for the RVFV L reference detection is linear to a Ct of 37 with an *R*-value of 0.9995 (Fig. 1, open circles)**,** where 55% of the RT-qPCR replicates are positive (5/9). The standard curve regression line for the RVFV M reference detection is linear to a Ct of 37 with an *R*-value of 0.9996 (Fig. 1, closed circles). The overall low Ct variability of RT-qPCR replicates even at low RNA copy number (standard deviation of less than 1 Ct), the linearity of the dynamic range of the assay, and the high analytic sensitivity of this multiplex RT-qPCR detection assay allows for accurate CN calculation at very low CNs (approximately 3–5 RNA copies).

**TABLE 2:** Quantitative RVFV RT-qPCR using *in vitro* transcribed RNA for analytical sensitivity and copy number determination for the RVFV M and L gene segment assays^*a*^

RT-qPCR M			RT-qPCR L		
M IVT RNA CN	Master mix A mean CT (STD)	Master mix B mean CT (STD)	L IVT RNA CN	Master mix A mean CT (STD)	Master mix B mean CT (STD)
26,000	17.7 (0.21)	NP	340,000	NP	NP
2,600	21.1 (0.14)	23.4 (0.18)	34,000	20.8 (0.19)	19.5 (0.15)
260	24.7 (0.50)	27.0 (0.18)	3,400	24.3 (0.13)	23.2 (0.18)
26	28.0 (0.69)	29.7 (1.0)	340	27.7 (0.23)	28.8 (0.14)
**2.6**	31.8 (0.95)	**35.4** (**1.0**)	34	31.2 (0.25)	30 (0.26)
**0.26**	**35.3** (**0.07**)	36.2 (0.5) 5/9^*b*^	**3.4**	**34.7** (**0.60**)	**36.3** (**0.67**)
0.026	37 (1/9^*b*^)	ND	0.34	37.8 (1.0) 5/9^*b*^	39.7 (0.53) 4/9^*b*^
0.026	ND	NP	0.34	ND	ND

^*a*^ Serial 10× dilutions of quantified IVT RNA were tested using a total of nine PCR replicate wells performed as three scientific replicates to evaluate the dynamic range of the RT-qPCR assays using the CFX thermocycler. This testing was performed side-by-side using two commercially available, one-step RT-qPCR master mixes: A, Quanta BioSciences; B, Applied Biosystems. Single-plex RT-qPCR data are reported; similar results were seen when assays were performed in multiplex (data not shown). CN, RNA copy number per reaction.

^*b*^ Replicate wells positive if not all were positive. STD, standard deviation; ND, not detected; NP, not performed. LOD_100_ numbers are in bold.

### Translation and evaluation of the RVFV M and L gene detection assays to RT-iiPCR

The detection of both the RVFV L and M gene segments in clinical samples offers high specificity and the ability to detect virus reassortment using a multiplex RT-qPCR detection assay (11). Therefore a two-segment multiplex detection strategy was employed for the development of the RT-iiPCR assay. Primer and probe sequences for single-segment detection assays are provided in Table 1. For the RT-iiPCR platform, the same conserved gene regions within the RVFV L and M segments were targeted, but modifications to primer/probe oligonucleotides were necessary for optimal performance of the RT-iiPCR assays on the portable platform. Oligonucleotide modifications include (i) probe modifications, (ii) primer modifications, and (iii) reduction of amplicon size for both RT-iiPCR assays (RVFV M and L). All primer and probe modifications were designed, tested and optimized for RT-iiPCR by GeneReach prior to lyophilization and prior to testing using IVT RNA or RNA derived from virus stocks or clinical samples.

### Analytical sensitivity of RVFV L and M segment detection assays on POCKIT device and establishment of quantitative *in vitro* transcribed RNA controls

Table 3 provides comparative analytical sensitivity for the detection of the RVFV RT-iiPCR L and M segments on the POCKIT in a side-by-side evaluation with the reference RT-qPCR assay utilizing quantified IVT RNA. RT-qPCR Ct results are directly comparable to the plus/minus detection of the POCKIT since equal molar ratios of RNA are present in the RT-qPCR and the RT-iiPCR reaction tubes. In three test replicates, the POCKIT device detected 0.26 RNA copies and 3.4 RNA copies per reaction for RVFV M and L RNA, respectively. The performance evaluation of the POCKIT using IVT RNA demonstrated equivalent analytical sensitivity to the RT-qPCR reference assay and the ability of the portable device to detect various concentrations of targets accurately and with good reproducibility using IVT RNA. The supplemental data provided in Table S1 further demonstrate the reproducibility of detecting the RVFV L and M segments on the POCKIT. Table S1 summarizes the results of testing randomized dilutions of IVT RNA side-by-side using the RT-qPCR and RT-iiPCR assays. This panel of samples mimics a proficiency panel that is utilized as training and reagent testing for end users in diagnostic settings. Similar training panels provided by either the USDA or the CDC exist for other regulated pathogens in the United States and serve as a means for reagent and personnel evaluation using nucleic acids from a non-infectious source. Utilizing variable RNA concentrations over the quantitative range of the RT-PCR assays and testing blindly in triplicate, side-by-side on both platforms, the RT-iiPCR reagents in the PON device demonstrate 100% sensitivity, specificity, and reproducibility. Similar results were obtained when a different person performed the testing independently, when the samples (RNA in lysis buffer) underwent automated magnetic bead extraction on the TacoMini (GeneReach, USA), or when RNA was re-tested using either master mix A or B in the reference assay (data not shown). Importantly, this testing also includes randomized “no RNA” samples (#5, 10, and 14), in addition to the IVT RNA and RT-qPCR controls. These negative samples established the means to evaluate the potential for RNA contamination when performing an evaluation of highly sensitive RT-qPCR detection methods using moderate to high RNA copy samples (#1, #7, and #15). During the development and performance testing of the proficiency panel, no errant Ct data (false positives due to contamination) were detected nor were any RT-iiPCR replicates erroneous reported as positive.

**TABLE 3:** Analytical sensitivity for the detection of RVFV L and M gene segments using RT-iiPCR assays on the portable device, POCKIT, and the reference multiplex RT-qPCR assay performed on the laboratory thermocycler using *in vitro* transcribed RNA^*a*^

Gene target	RT-qPCR/Bio-Rad CFX thermocycler			RT-iiPCR/POCKIT
	CN	Ct	Positive/tested (%)	Positive/tested (%)
RVFV-M	2,600	21.16	9/9 (100)	3/3 (100)
	260	24.75	9/9 (100)	3/3 (100)
	26	28.14	9/9 (100)	3/3 (100)
	2.6	31.75	9/9 (100)	3/3 (100)
	**0.26**	**35.08**	**9/9 (100**)	**3/3 (100**)
	0.026	36.46	1/9 (11)	0/3
RVFV-L	3,400	24.23	9/9 (100)	3/3 (100)
	340	27.78	9/9 (100)	3/3 (100)
	**3.4**	**34.87**	**9/9 (100**)	**3/3 (100**)
	0.34	37.16	5/9 (55)	2/3 (66)
	0.034	ND	0/9 (0)	0/3 (0)

^*a*^ Data presented utilized master mix A. Master mix A was selected as the primary master mix for the remainder of the study. CN, IVT RNA copy number per reaction; Ct, mean cycle threshold for PCR detection; %, percent of positive replicates. LOD_100_ numbers are in bold.

### Determination of analytical sensitivity for the detection of RVFV M and L RNA for RT-qPCR and RT-iiPCR on the POCKIT using viral RNA isolated from virus stocks

Table 4 summarizes analytical sensitivity for the detection of RVFV M and L RNA using RT-iiPCR on the POCKIT with RNA purified from two RVFV vaccine strains (MP-12 and Smithburn) and two virulent wild-type strains (Ken06 and SA01). Viral RNA was purified using automated magnetic bead extraction protocols. Tenfold serial dilutions of viral RNA from RVFV strains Smithburn, MP-12, Ken06, and SA01 were tested side-by-side with RT-iiPCR on the POCKIT and the RT-qPCR reference assays. RT-qPCR master mixes A and B were also tested, and RT-qPCR was performed using nine RT-qPCR replicates while iiRT-PCR used three RT-qPCR replicates. RNA CN was calculated by a relative standard curve methodology. The analytical sensitivity (LOD_100_) for M and L RNA detection using RT-iiPCR on the POCKIT was equivalent to RT-qPCR reference assay. Comparative data using master mix A and the RT-iiPCR results are summarized in Table 4 for all virus strains tested. Smithburn RNA was only tested with master mix B at the Kenya site. The analytical sensitivity (LOD_100_) for M and L RNA detection for both PCR platforms using is MP-12 is 2.4 and 150 RNA copies per reaction, and 1.5 and 120 RNA copies per reaction using Smithburn vaccine strains. The analytical sensitivity for both PCR platforms for M and L RNA detection of RVFV wild-type viruses, Ken06 and SA01, was 4 and 6.3 RNA copies per reaction and 10 and 6.3 copies per reaction, respectively. In some instances, iiRT-PCR was superior in the detection of RVFV RNA when compared to RT-qPCR. During the testing, no false positives were detected in either the RT-qPCR or RT-iiPCR assays. NTC tests were performed at a rate of one per 15 test wells for RT-qPCR and one per 6–7 RT-iiPCR test wells.

**TABLE 4:** Analytical sensitivity for the detection of RVFV L and M gene segments using viral RNA. Performance of the RT-iiPCR assays on the portable device, POCKIT, was compared to the reference multiplex RT-qPCR assay performed on the laboratory thermocycler using viral RNA^*a*^

	RT-qPCR on laboratory thermocycler				RT-iiPCR on POCKIT	
**A. MP12**	M RT-qPCR		L RT-qPCR		M RT-iiPCR	L RT-iiPCR
**CN (M/L)**	Ct	+/tested (%)	Ct	+/tested (%)	+/tested (%)	+/tested (%)
9/38	28	9/9 (100)	29.6	9/9 (100)	3/3 (100)	3/3 (100)
15	31.7	9/9 (100)	33.4	9/9 (100)	3/3 (100)	3/3 (100)
**1.5/2.4**	**35.3**	**9/9 (100)**	**36.8**	**9/9 (100)**	**3/3 (100)**	**2/3 (66)**
0.15/1.7	38	7/9 (78)	39.2	1/9 (11)	2/3 (66)	1/3 (33)
0.02/0	38.9	1/9 (11)	ND	0/9 (0)	0/3 (0)	ND
		
**B. Smithburn**	M RT-qPCR		L RT-qPCR		M RT-iiPCR	L RT-iiPCR
**CN (M/L)**	Ct	+/tested (%)	Ct	+/tested (%)	+/tested (%)	+/tested (%)
15,000/12,000	20.8	3/3 (100)	21.9	3/3 (100)	3/3 (100)	3/3 (100)
1,500/1,200	24.9	3/3 (100)	26.8	3/3 (100)	3/3 (100)	3/3 (100)
**150/120**	**28.2**	**3/3 (100)**	**30.5**	**3/3 (100)**	**3/3 (100)**	3/3 (100)
15/12	32.6	2/3 (66)	36.3	2/3 (66)	3/3 (33)	**3/3 (100)**
1.5/1.2	35.7	1/3 (33)	ND	1/3 (33)	0/3 (0)	0/3 (0)
		
**C. Kenya 06**	M RT-qPCR		L RT-qPCR		M RT-iiPCR	L RT-iiPCR
**CN (M/L)**	Ct	+/tested (%)	Ct	+/tested (%)	+/tested (%)	+/tested (%)
514/1,403	28.2	9/9 (100)	26.7	9/9 (100)	3/3 (100)	3/3 (100)
39/132	31.6	9/9 (100)	29.8	9/9 (100)	3/3 (100)	3/3 (100)
10/4	34.9	9/9 (100)	33.3	9/9 (100)	3/3 (100)	3/3 (100)
**1.5/1.5**	**38**	**9/9 (100)**	**36.8**	**9/9 (100)**	3/3 (100)	**3/3 (100)**
0.15/0.15	39	3/9 (33)	38.1	2/9 (22)	**3/3 (100)**	ND
0.02/0.02	ND	0/9 (0)	ND	0/9 (0)	1/3 (33)	ND
		
D. SA 01	M RT-qPCR		L RT-qPCR		M RT-iiPCR	L RT-iiPCR
**CN (M/L)**	Ct	+/tested (%)	Ct	+/tested (%)	+/tested (%)	+/tested (%)
14,000	23.8	9/9 (100)	21.9	9/9 (100)	3/3 (100)	3/3 (100)
1,310	27	9/9 (100)	25.7	9/9 (100)	3/3 (100)	3/3 (100)
63	31	9/9 (100)	29.2	9/9 (100)	3/3 (100)	3/3 (100)
**6.3**	**34.1**	**9/9 (100)**	**32.6**	**9/9 (100)**	**3/3 (100)**	3/3 (100)
1.5	37	7/9 (78)	35.4	7/9 (78)	1/3 (33)	**3/3 (100)**
0.15	38.3	3/9 (33)	37.1	4/9 (36)	0/3 (0)	1/3 (33)

^*a*^ RVFV strains utilized include A. MP12 and B. Smithburn (vaccine strains); C. Kenya 06 (128B) and D. Saudi Arabia 2001 (SA 01) (virulent strains). Limits of detection above 50% positive are bolded. CN, RNA copy number per reaction; Ct, mean cycle threshold for RT-qPCR detection (*n* = 9 except Smithburn where *n* = 3). RT-iiPCR was performed in triplicate; %, percent positive. LOD_100_ numbers are in bold.

**TABLE 5:** Clinical sensitivity and specificity detection using RVFV L and M specific RT-iiPCR assays on the portable device, POCKIT, using sera from RVFV experimentally infected cattle and sheep; cohort 1

		RT-qPCR		Total tested	Sensitivity^a^	[95% CI]^a^	Specificity (%)^a^	[95% CI]^a^	Kappa#	[95% CI]^a^
Species/RVFV segment	iiRT-PCR on POCKIT				(%)					
		Positive	Negative							
Sheep M	Positive	55	3	104	96.5	[86.8–99.4]	93.6	[81.4–98.3]	0.9	[0.82–0.99]
	Negative	2	44							
Sheep L	Positive	54	2	106	98.2	[89–100]	96.1	[85.4–99.3]	0.94	[0.88–1]
	Negative	1	49							
Cattle M	Positive	60	2	113	92.3	[82.2–97.1]	95.8	[84.5–99.2]	0.87	0.78–0.96
	Negative	5	46							
Cattle L	Positive	44	1	94	88	[75–95]	97.7	[87–100]	0.85	0.75–0.96]
	Negative	6	43							
Sheep dual (L/M)	Positive	52	0	100	96.3	[86.1–99.4]	100	[90.4–100]	0.96	[0.90–1]
	Negative	2	46							
Cattle dual (L/M)	Positive	39	0	81	90.7	[77–97]	100	[88.6–100]	0.9	[0.81–0.996]
	Negative	4	38							
All (L/M)	Positive	91	0	181	93.8	[86.5–97.5]	100	[95–100]	0.93	[0.88–0.98}
	Negative	6	84							

^a^ Sensitivity, specificity, confidence internals (CIs), and kappa were calculated using Vassar (http://vassarstats.net/).

### Evaluation of the clinical sensitivity and specificity for RVFV M and L segment detection in ruminant sera using RT-iiPCR on the POCKIT

The clinical sensitivity and specificity of the single-plex (RVFV M or L) and dual detection of the M and L gene segments RT-iiPCR using RVFV RNA purified from clinical samples were determined using serum from four animal cohorts: (i) sera from domestic cattle and sheep that had either been mock-infected or experimentally infected with wild-type, virulent RVFV, (ii) archived serum from RVFV-negative domestic goats, (iii) fresh and archived field sera collected from sheep, cattle, and goats in Kenya, and (iv) archived field sera collected from sheep, cattle, goats, and two antelope species [impala (*Aepyceros melampus*) and nyala (*Tragelaphus angasii*)] in South Africa.

The first cohort (cohort 1) consisted of TRIzol-inactivated serum samples collected from sheep (*n* = 13) and cattle (*n* = 15) experimentally infected with two wild-type RVFV strains (Ken06 and SA01) (13, 14). Sera from two mock-infected control animals per group (*n* = 4 total) were included in the testing and analysis. Data in Table 5 summarize the results of RT-qPCR testing of sera collected from day 0 up to day 6 post-infection for sheep and cattle. Multiplex RT-qPCR was performed on the laboratory thermocycler as the reference standard for the RT-iiPCR, and the cut-off value for reporting positivity was 38 Ct. Samples with RT-qPCR Ct at or below the cut-off (positive) or those with no Ct detected (negative) were included in the performance analysis for RT-iiPCR. Samples with a Ct above the cut-off are classified as suspect/negative and were not included in the analysis since the RT-iiPCR is a non-quantitative plus/minus detection.

Sensitivity and specificity (Sen/Spe) of detection of individual targets, RVFV M or RVFV L RNA, in cattle and sheep were determined independently. This analysis was followed by Sen/Spe analysis for each host species followed by the determination of the overall Sen/Spe for RVFV detection using the dual (M/L) RNA detection approach, similar to the current recommended use of the reference multiplex RT-qPCR assay.

The detection of individual RVFV RNA segments for each species (cattle or sheep) was analyzed independently and is summarized in Table 5. For sheep, the sensitivity and specificity of individual RVFV M and L RNAs are similar at 96.5% and 98.2% (Sen) and 93% and 96.1% (Spe), respectively. For cattle, the sensitivity of individual RVFV M and L RNAs is slightly reduced at 92.3% and 88%, respectively, while specificity is high at 95.8% and 97.7%, respectively. The slightly reduced sensitivity for M and L segment detection in cattle is due to the lower viral load of RVFV found in cattle during acute viremia as compared to sheep (data not shown). This is particularly true for the L segment detection assay and for cattle infected with the RVFV SA01 strain. The data highlight the biological variability seen with various wild-type RVFV strains in different host species.

A dual-segment RT-PCR detection strategy offers the highest confidence for specific and sensitive detection of RVFV (11). For the present analysis, positive clinical samples are defined when the M and L segment assays meet the RT-qPCR-determined Ct cut-off of ≤38 Ct. The data for the dual RT-qPCR assay are summarized in the third panel of Table 5. For sheep, the sensitivity for dual-segment detection using RT-iiPCR is similar to the single-plex M RNA detection at 96.3%, suggesting that M RNA detection is more sensitive than L RNA detection in infected animals, as was observed in the LOD determination for each assay. For cattle, the overall sensitivity for dual RVFV segment detection using RT-iiPCR is 90.7% when detecting both gene segments. Dual-RNA segment detection in cattle and sheep results in improved specificity at 100% since both segments need to be detected to define a positive result. Overall, the sensitivity and specificity for dual-RVFV segment detection by RT-iiPCR on the POCKIT in sheep and cattle experimentally infected with RVFV with two virulent RVFV strains were 93.8% and 100%, respectively. The data suggest that the dual-segment RT-iiPCR detection assay is a viable strategy for RVFV detection in the field.

To confirm the specificity of the detection of RVFV RNA using the portable device and prior to testing field sera from endemic countries, we included testing archived serum samples from domestic goats (*n* = 50) originating from the United States (cohort 2). Sera from this cohort were tested side-by-side using the RT-qPCR reference assays on a laboratory thermocycler and the RT-iiPCR on the POCKIT for RVFV M and L segment detection. False-positive signals were not seen using the RT-qPCR reference assays and the RT-iiPCR on the POCKIT, demonstrating 100% specificity using known negative field sera.

The clinical sensitivity for the detection of RVFV RNA using RT-iiPCR assays on the POCKIT as compared to multiplex RT-qPCR assays using field sera collected in Africa is summarized in Table 6. Data shown represent serum collections from two site visits at different time points: Kenya in spring 2016 (cohort 3) and South Africa in the fall of 2017 (cohort 4). Cohort 3 consisted of mixed cattle, sheep, and goat samples (*n* = 97) that were collected during a minor RVFV outbreak event and during a vaccination control program. Cohort 4 consists of mixed cattle, sheep, goat, and antelope sera (n=96) collected during a period of low but detectable seroprevalence of RVFV IgM antibodies. Data are reported for RVFV M and L RNA dual detection. A total of 193 livestock sera tested side-by-side with the RT-qPCR and RT-iiPCR assays from two RVFV-endemic countries produced two positive samples for both RVFV segments on both platforms, resulting in a specificity of 100% for all field samples tested in Africa. Although the number of positive samples in this study is low, the data coupled with the testing of samples from experimentally infected sheep and cattle demonstrate exceptional performance for the RNA segment RVFV detection assay on the field-deployable device, the POCKIT.

**TABLE 6:** Clinical sensitivity for the detection of RVFV using RT-iiPCR assays on the portable device, POCKIT, as compared to the multiplex RT-qPCR assay using field serum collected in Africa^*a*^

Cohort	iiPCR on POCKIT	RT-qPCR		Total tested	Sensitivity (%)	[95% CI]	Specificity (%)	[95% CI]	Kappa	[95% CI]
		Positive	Negative							
Cohort 3: Kenya RVFV L/M	Positive	1	0	97	100	[95–100]	100	[95–100]	1	[0]
	Negative	0	96							
Cohort 4: South Africa RVFV L/M	Positive	1	0	96	100	[95–100]	100	[95–100]	1	[0]
	Negative	0	95							

^*a*^ Data represent serum collections from two site visits: Kenya spring 2016 (cohort 3) and South Africa fall 2017 (cohort 4). Cohort 3 consists of mixed cattle, sheep, and goat samples (*n* = 97). Cohort 4 consists of mixed cattle, sheep, goat, and wild ruminant sera collected during low-level sero-prevalence of RVFV IgM antibodies and suspect RVFV infections in domestic ruminant populations (*n* = 96). Wild ruminant species (antelope and ibex) sera originated from South Africa and eastern African countries. Data are reported for L and M dual detection.

## DISCUSSION

RVFV infection in domestic ruminants and humans clinically mimics many other viral and bacterial infections, and during outbreaks, the occurrence of disease in animals generally precedes human cases. Therefore, rapid detection of RVFV in animals is a feasible strategy for the early detection of an outbreak and will help prevent human cases. In the present study, the excellent analytical sensitivity of the RT-iiPCR assay performed on a deployable device, POCKIT, for the detection of both synthetic and viral-derived RNA was demonstrated. These field deployable PCR assays were performed using lyophilized RT-iiPCR mixes stable at room temperature on a portable nucleic acid extraction machine amenable for PCR testing in field conditions. In parallel, RT-iiPCR analyses were performed side-by-side using two WOAH-approved RVFV RNA detection assays performed as a multiplex reference assay on the laboratory thermocycler (https://www.woah.org/fileadmin/Home/eng/Health_standards/tahm/3.01.18_RVF.pdf). The analytical sensitivity and LOD_100_ for RVFV M and L RNA on the POCKIT were equivalent to that of the RT-qPCR multiplex reference assay, with detection of approximately 0.1–3 copies/reaction for RVFV M gene segment RNA and 1–3 copies for the L gene segment detection. These results suggest that when testing clinical samples for RVFV RNA, the performance of RT-iiPCR on the POCKIT should be comparable to laboratory-based RT-qPCR detection particularly at RNA copy numbers greater than 10 per reaction. Moreover, these results suggest that confident detection of RVFV M and L RNA in clinical samples in the field would be feasible (11).

When testing archival samples from sheep and cattle experimentally infected with two virulent strains of RVFV (SA01 and Ken06), the overall sensitivity for the detection of RVFV M and L RNA gene segments RNA using POCKIT was 100% and 93.8%, respectively. There was reduced sensitivity of PCR detection of RVFV RNA from cattle, particularly those infected with RVFV strain SA01. This is attributed to the lower viral loads associated with RVFV SA01 in ruminants, which is most profound in cattle. Average RT-qPCR RVFV M and L Ct values at peak viremia (D2, *n* = 12) were 27.5 for sheep infected with SA01 and 22.5 for those infected with Kenya 06. In contrast, peak viremia in cattle occurred at D2 in 4 of 10 animals or at D3 for 6 of 10 animals, with a mean Ct of 34 for cattle infected with SA01 and mean Ct of 27 for cattle infected with Kenya 06. This equates to approximately 2–3 logs less SA01 viral RNA present in both ruminant species when compared to Kenya 06. In this study, samples that were missed when tested with RT-iiPCR were obtained from SA01-infected cattle with RT-qPCR Cts of greater than 35. Moreover, when RT-iiPCR missed RVFV detection in sheep samples, the RT-qPCR Cts were greater than 35. An RT-qPCR Ct of 35–38 is near or at the LOD_50_ of the M and L RT-qPCR assays and equates to less than single-copy detection of M RNA or approximately 1–3 copy detection of L gene segment RNA (Table 1). However, the detection of greater than 3–5 copies (Ct of 35 or less) of viral RNA gives high confidence in the performance of the assay for field testing of animals during the acute stage of infection when animals display clinical disease and have the highest viral loads. During the acute phase of RVF (2–3 DPC), the sensitivity for RT-iiPCR detection on POCKIT was 100% in samples from experimentally infected sheep and cattle. Even with low copy RNA or intermittent viremia, there is a reasonable expectation that infected animals would be detected in the field using a portable automated nucleic extraction device and the deployable POCKIT provided that a sufficient number of animals would be tested. The testing of two cohorts of clinical samples in two endemic African countries, unfortunately, could not be performed during a large RVF outbreak, resulting in only 2 of 193 samples testing positive for RVFV RNA (M and L gene detection). Additionally, during this testing, we evaluated the feasibility and performance of the portable magnetic bead processor, TacoMini, (GeneReach, USA), for rapid automated nucleaic acid purification. This insturment can process eight samples per run in a mobile laboratory or the field. This instrument was easy to use and performed exceptionally well in a mobile laboratory setting. Since it was not within the scope of the study, we did not validate the extraction protocol allowing for inactivation of RVFV during purification of viral RNA. Ongoing work adresses virus inactivation during the RNA extraction procedure on the TacoMini. Results obtained in these studies demonstrate a sensitivity and specificity of RT-iiPCR technology that is equivalent to the multiplex RT-qPCR assay using two RT-qPCR assays recommended by the WOAH for RT-qPCR detection of RVFV in sera from ruminants.

Previous technology evaluated for the detection of RVFV in a field setting includes the use of gold nanoparticles for the identification of non-amplified RVFV RNA with a reported LOD of 10 RNA copies/reaction (16), and reverse transcription loop-mediated isothermal amplification (RT-LAMP) with a reported LOD of 10 RNA copies per reaction of the RVFV L gene segment (17). Additionally, recombinase polymerase amplification (RPA) of the S segment RNA has been reported to have a LOD of 19 copies per reaction (18). Although these technologies offer promising solutions for field detection of RVFV in biologicals, each has drawbacks aside from only detecting one gene segment of the RVFV genome. Gold particles can bind DNA and RNA from other pathogens and the host, reducing the sensitivity of detection by overloading binding sites and leading to false-positive results. Moreover, in some instances, it can be challenging to design primers for RT-LAMP and RPA assays that are adequately specific and sensitive for small viral RNA fragments. Moreover, DNA amplification using the RT-LAMP and RPA very rapidly creates massively high copy number of amplicons that can easly result in environmental contamination and false positives during field testing. The POCKIT and RT-iiPCR technology evaluated here uses proven qPCR amplification and detection methodology in a novel format: convection PCR that occurs in a tightly closed capillary tube system, and provided that good molecular laboratory practices are followed during testing, this technology should not result in environmental contamination. Lastly, the use of lyophilized RT-iiPCR reagents (primers, probes, and enzymes) has great advantages in field situations where conditions for cold storage and molecular work are far from ideal. Using lyophilized reagents, we are able to attain sensitive and specific amplicon detection using sequence-specific TaqMan probes in the preprogrammed POCKIT device. The device can run eight reactions at a time allowing for the use of a two-gene detection format and the testing of multiple samples, such as is reported here, for high-confidence pathogen detection.

In summary, a sensitive and specific multigene detection method for RVFV was developed for RT-iiPCR on the POCKIT device. On the basis of equivalent sensitivity and specificity as compared to WOAH-approved reference assays performed in a specalized laboratory, the system merits consideration for deployment in the field to support preparedness protocols in countries at risk of RVF outbreaks or virus introduction and for surveillance of virus activity in succeptable species. The portable iiPCR technology will facilitate the implementation of control and risk assessment measures in endemic countries to prevent animal and human disease and could be added to diagnostic stockpiles to rapidly respond to a RVF outbreak.

## AFTER EPUB

*[This article was published on 2 February 2024 with an incorrect affiliation for Peter Thompson. The affiliation was corrected in the current version, posted on 7 February 2024.]*

## SUPPLEMENTAL MATERIAL

Table S1 jcm.00430-23-s0001.pdf RVFV reference panel performance and reproducibility.

ASM does not own the copyrights to Supplemental Material that may be linked to, or accessed through, an article. The authors have granted ASM a non-exclusive, world-wide license to publish the Supplemental Material files. Please contact the corresponding author directly for reuse.
