# Supplementary material for: Rift Valley Fever virus M and L genome segment detection: a comparison of field-deployable reverse transcription insulated isothermal PCR (RT-iiPCR) and laboratory-based multiplex reverse transcription real-time PCR
Source: J Clin Microbiol. 2024 Feb 2;62(3):e00430-23. doi: 10.1128/jcm.00430-23 (PMC10935642; doi:10.1128/jcm.00430-23)
Supplement: Table S1 — RVFV reference panel performance and reproducibility. [file jcm.00430-23-s0001.pdf]

Table S1: RVFV Reference Panel Performance and reproducibility for the detection of the RVFV L and M segments using RT-iiPCR assays on the portable device, POCKIT^{TM}, compared to the reference multiplex RT-qPCR assay performed on a laboratory thermocycler using IVT RNA for the L and M segments).

Sample	RT-qPCR on laboratory thermocycler		RT-iiPCR on POCKIT^{TM}	
	M RT-qPCR Ct	L RT-qPCR Ct	M RT-iiPCR	L RT-iiPCR
1	21.1	20.7	3/3	3/3
3	24.73	24.6	3/3	3/3
2	28.34	25.1	3/3	3/3
4	32.16	27.9	3/3	3/3
5	ND	ND	ND	ND
6	25.18	24.2	3/3	3/3
7	21.4	21.3	3/3	3/3
8	28.09	27.4	3/3	3/3
9	30.48	21.2	3/3	3/3
10	ND	ND	ND	ND
11	25.10	24.0	3/3	3/3
12	28.22	27.5	3/3	3/3
13	32.63	28.0	3/3	3/3
14	ND	ND	ND	ND
15	21.3	21.9	3/3	3/3

Randomized serial dilutions of RVFV M and L IVT RNA (and 3 “no RNA” controls, were subjected to RT-qPCR and RT-iiPCR using triplicate PCR wells.
Ct= mean cycle threshold for RT-qPCR detection**;** ND=not detected
